# Acceptability and Barriers to Chronic Pain Treatment in Refugee Torture Survivors

**DOI:** 10.1001/jamanetworkopen.2025.29775

**Published:** 2025-08-28

**Authors:** Sargun Kaur Virk, Samantha Tham, Claudia Hatef, Tanzilya Oren, Lola Berger, Adam Tucker, Andrew Robert Milewski, Inmaculada de Melo-Martin, Gunisha Kaur

**Affiliations:** 1 Department of Anesthesiology, Weill Cornell Medicine, York Avenue, New York, New York; 2 Weill Cornell Medicine Medical College, New York, New York; 3 SUNY Downstate College of Medicine, Brooklyn, New York; 4 NYC Department of Health and Mental Hygiene, New York, New York; 5 Division of Medical Ethics, Weill Cornell Medicine, York Avenue, New York, New York

## Abstract

**Importance:**

Chronic somatic pain is prevalent in refugee torture survivors, yet it remains underdiagnosed and undertreated. Understanding the acceptability of and barriers to treatment is essential for facilitating access and improving care in this population.

**Objective:**

To assess the acceptability of treatment for chronic somatic pain and to identify factors influencing treatment access among refugee torture survivors.

**Design, Setting, and Participants:**

This qualitative study was conducted between September 20, 2021, and December 20, 2023. Interviews were thematically analyzed using a framework guided by the Gelberg-Andersen Behavioral Model of Healthcare Utilization for Vulnerable Populations. This study was conducted through the Weill Cornell Center for Human Rights (WCCHR), a large academic, medical-legal human rights center in the US serving a globally representative population of refugees. Adult refugee torture survivors aged 18 years or older with chronic pain as diagnosed by a specialist pain physician were included. Data were examined from December 2023 to October 2024.

**Exposures:**

Participants were asked about their attitudes toward 3 standard treatment modalities for chronic somatic pain, including nonopioid analgesics, trigger-point injections, and physical therapy.

**Main Outcomes and Measures:**

Pain treatment acceptability and perceived barriers to treatment were categorized using the Andersen Model.

**Results:**

The 25 participants had a mean (SD) age of 37 (9.3) years, 18 (72%) were male, and 25 (100%) were asylum seekers representing 5 low-income United Nations regions. All participants were receptive to at least 1 of the proposed treatment modalities. Acceptability was primarily influenced by predisposing and need factors, including trust in the health care systems and a strong motivation to alleviate pain. However, multiple barriers impeded treatment uptake. These barriers were categorized as predisposing factors (eg, language barriers, cultural differences, and immigration status), enabling factors (eg, perceived inadequacies in insurance coverage, financial and work-related constraints, and logistical challenges), and need factors (eg, pain severity and the compounded stress of legal status).

**Conclusions and Relevance:**

Although refugee torture survivors are open to chronic pain treatment, this qualitative study suggests that systemic barriers hinder use. Addressing these barriers is essential for improving health care access in this vulnerable population.

## Key Points

**Question:**

Which chronic somatic pain treatment modalities are acceptable to refugee torture survivors, and what factors influence their access to these treatments?

**Findings:**

In this qualitative study of 25 refugee torture survivors, all participants expressed willingness to engage in treatment driven by a trust in the healthcare systems and a strong desire to alleviate pain. Despite high acceptability, multiple barriers hindering access to treatment were reported, including language, cultural differences, immigration status, financial constraints, and logistical challenges.

**Meaning:**

These findings suggest that although refugee torture survivors are willing to engage in chronic pain treatment, structural and systemic barriers limit their ability to do so.

## Teaser

This qualitative study examines the acceptability of treatment for chronic somatic pain and identifies factors influencing treatment access reported by refugee torture survivors.

## Introduction

The World Medical Association (WMA) Declaration of Tokyo defines torture as the “deliberate, systematic or wanton infliction of physical or mental suffering by 1 or more persons acting alone or on the orders of any authority, to force another person to yield information, to make a confession, or for any other reason.”^1^ Up to 44% of refugees living in the US have experienced torture, suggesting that at least 1.3 million torture survivors reside in the US.^2^ With a prevalence of 78% to 83%, pain and pain-related disabilities are among the most common sequelae of torture.^3,4,5^ Nevertheless, chronic pain remains largely underdiagnosed and undertreated in this population: less than 16% of affected individuals receive accurate diagnoses and less than 10% receive treatment.^6^

Compounded by psychological harm, migration-associated trauma, and preexisting medical conditions, the pain experience of torture survivors is complex. Although evidence for appropriate pain interventions is poor, treatment modalities that have been studied include pharmacological analgesia, physical rehabilitation, and talk therapies.^7^ Because some health professionals, such as prison doctors, have been complicit in torture, treating chronic pain in torture survivors might require considering the potential for distrust of health professionals.^8,9,10^ Moreover, certain pain treatment modalities that could resemble aspects of previous torture experiences, such as those requiring venipuncture or scans performed in closed spaces, may be intolerable to torture survivors.^11^ Preexisting legal, social, and welfare challenges present additional obstacles to seeking health care in this population.^12^

To date, exploration of torture survivors’ perspectives on the acceptability of pain treatments and experiences interfacing with health care systems is scarce.^13,14,15^ We applied the Andersen Behavioral Model^16^ to explore contextual and individual factors influencing the acceptability of somatic pain treatment among torture survivors. The model has 3 core domains: predisposing factors (age, sex, marital status, education, race or ethnicity, occupation, and beliefs), enabling factors (accessibility and availability of family and community resources), and need factors (individual’s perceived and evaluated functional abilities, symptoms, and general health condition). The Andersen framework has been adapted for vulnerable groups, including immigrants.^17,18^ Identifying both individual and contextual factors that contribute to the underuse of health services to treat torture survivors’ pain can inform future decisions on the most appropriate treatment modalities. Therefore, we aimed to qualitatively assess the acceptability of 3 standard treatment modalities for chronic somatic pain (ie, nonopioid analgesics, trigger-point injections, and physical therapy) and to elucidate factors associated with access to chronic somatic pain treatment in torture survivors.

## Methods

### Study Design

The Gelberg and Andersen Behavioral Model of Health Care Utilization for Vulnerable Populations was used as a conceptual framework for conducting qualitative interviews of torture survivors with chronic pain.^18^ The interviews assessed (1) participants’ understanding and acceptance of 3 standard chronic somatic pain interventions (nonopioid analgesics, trigger-point injections, and physical therapy) and (2) the biosocial context of migration and current living conditions. The 3 treatment modalities were selected in consultation with pain specialists as first line treatments for chronic pain. The research was approved by the Weill Cornell institutional review board and registered at ClinicalTrials.gov. Participants provided informed consent in their primary language, and interpreters were used as necessary. Data were recorded in case report forms and stored in REDCap.

All authors are responsible for the accuracy and completeness of the data and analysis and for maintaining the study’s fidelity to the protocol. This study followed the Standards for Reporting Qualitative Research (SRQR) reporting guideline.^19^

### Study Population

Eligible participants were adult refugee torture survivors (aged 18 years or older), as defined by the WCM Declaration of Tokyo.^1^ Participants diagnosed with a chronic pain condition by a specialist pain physician were included. Participants were recruited consecutively through the Weill Cornell Center for Human Rights (WCCHR), the largest academic, medical-legal human rights center in the US that serves a globally representative population.

The study was conducted from September 20, 2021, to December 20, 2023. The semistructured interviews, which lasted approximately 30 to 45 minutes, were audio-recorded with participants’ consent, and subsequently transcribed and translated into English.

### Data Analysis

Interviews were transcribed verbatim and culturally interpreted by a native language speaker. A qualitative deductive thematic analysis was conducted, guided by the Andersen Model using Dedoose version 8.1 (Dedoose).

Using this framework, a preliminary coding tree was developed a priori to categorize participant responses into predisposing, enabling, or need domains.^16^ After an initial review of all transcripts, 2 coders (T.O. and C.H.) independently generated major codes and child codes to develop preliminary themes. Through an iterative process, the coders compared, re-arranged, and renamed codes, added new codes and themes, and combined all initial codes into broader thematic categories. Three authors (T.O., S.V., and S.T.) agreed on the code tree with codes pertinent to the research questions and selected representative quotes.

The interrater reliability was tested in Dedoose, with a Cohen κ correlation coefficient value of 0.62, which denotes good reliability between the coders. To convey the relative prominence of themes, we used terms such as few, several, some, many, majority, most, almost all, and all to denote approximate thresholds (few, 2 to 4; several, 4 to 6; some, 6 to 10; many, 10 to 15; majority, 15 to 18; most, 18 to 22; almost all, 22 to 24; all, 25).

The research team included clinicians and researchers with experience working with refugee populations. To minimize bias, interviews followed a semi-structured guide, and 2 coders independently analyzed the transcripts using the Andersen Model. Discrepancies were resolved through discussion, and reflexive practices were used throughout to ensure themes reflected participant experiences. Data were analyzed from December 2023 to October 2024.

## Results

### Participant Demographics

This qualitative study included 25 participants with a mean (SD) age of 37 (9.3) years. The majority of participants were male (18 [72%]). English was the most commonly spoken language (10 [40%]), and the sample represented all 5 low-income United Nations regions, with the majority from Latin America and the Caribbean (11 [44%]). A total of 22 participants (88%) reported current or prior treatments for their chronic somatic pain. The number of individuals with previous experience with each of the 3 different treatment modalities is provided in Table 1.

**Table 1.** Participants’ Characteristics

Characteristic	Participants, No. (%) (N = 25)
Sex	
Male	18 (72)
Female	7 (28)
Country of origin	
Angola	1 (4)
Burkina Faso	2 (8)
Chad	1 (4)
China	1 (4)
Ecuador	1 (4)
El Salvador	2 (8)
Georgia	1 (4)
Ghana	1 (4)
Guatemala	1 (4)
Guinea	2 (8)
Honduras	4 (16)
India	1 (4)
Jamaica	2 (8)
Nicaragua	1 (4)
Nigeria	1 (4)
Russia	3 (12)
Age, y	
25-40	18 (72)
41-55	6 (24)
56-65	1 (4)
Interview language	
English	10 (40)
French	4 (16)
Mandarin	9 (36)
Spanish	1 (4)
Russian	1 (4)
Prior therapy used	
Nonopioid analgesics	15 (60)
Trigger-point injections	0
Physical therapy	4 (16)

Although 4 participants (16%) declined trigger point injections and 3 (12%) were not open to physical therapy, all participants expressed interest in at least 1 of the 3 treatment modalities—nonopioid analgesics, trigger-point injections, and physical therapy. Notably, while the majority of participants (15 [60%]) reported relying on nonopioid analgesics, none had received trigger-point injections. Although a few individuals (4 [16%]) had received physical therapy, numerous challenges hindered continued access to this treatment modality.

Within the domains advanced by the Andersen Model, key themes—including factors that facilitated and hindered uptake of chronic pain treatment—shaped participants’ perspectives of the 3 proposed treatment modalities: nonopioid analgesics, trigger point injections, and physical therapy. The themes—together with their associated subthemes and illustrative quotes—were organized according to and are presented within the Andersen Model domains of predisposing, enabling, and need factors (Table 2).

**Table 2.** Pain Treatment Acceptability Themes, Subthemes, and Representative Quotes

Domains	Themes and subthemes	Representative quotes (participant No.)
Predisposing	Trust in health care systems: health care clinicians and trusted environments	I would like to be seen by a specialist first…so, I can just go to a pharmacy and buy whatever they advise. (Participant 8)
	Concerns about pain treatment: effectiveness of pain treatment and experience or encounter with addiction	Tylenol, helps…after 8 hours…And then it comes back (Participant 6)
		If I think that I’m feeling pain, I’ll take a pill. I don’t mind taking pills, but I wouldn’t want to be addicted to any. (Participant 21)
	Language and cultural differences	Yeah, I’ve had an experience, and I felt so bad because it was of emergency to me but I couldn’t reach directly to the doctor. And the reception, was not able to understand my English very well. (Participant 6)
	Immigration status	Scared. Also Mr X said whoever used the benefits will not get green card, and it was huge. Right now, everybody already forgot about it, but it was a huge hit. Everybody actually that I know, immigrants, they said hell no, I’d rather die at home, especially the COVID and everything. Nobody wanted to even go. No, No. (Participant 17)
Enabling	Perceived inadequate insurance coverage	Well, if I had an emergency at home or at work, I guess I’d go to an emergency department at the hospital. But I’ve been there in the past and I say okay I’m here for a general check-up. But they always tell me that I need an insurance for that and then they tell me I don’t qualify. (Participant 8)
	Financial and work-related barriers: need to financially support dependents and unstable work conditions	Well, mainly my work you see because here we have to be very responsible. So also, if you’re here with a family, you have to take care of the family. Then so if I miss 1 or 2 days at work, it gets complicated because I get my expenses unbalanced. (Participant 11)
		Well, I don’t know if that’s done during the weekdays or how often because sometimes because of my work, it can get uncomfortable. (Participant 8)
	Logistical challenges	I will not be able to come because maybe what they give me and there is I can go out once in a month and then I need to go back. (Participant 14)
Need	Pain severity	When it hurts, it is excessively painful, and it is like all the nerves in my head hurts. (Participant 1)
	Strong desire to alleviate pain: open to all treatments and conditional acceptance based on pain severity	Yes [will be open to all]. As long as I can relieve the pain. (Participant 9)
		It depends on the level of pain. If the pain is severe, it may be useful. (Participant 15)
	Stress of legal status	Well, when I am very stressed out, when I am overthinking things, I tend to get something like a headache, but it is severe pain in my brain. Well, actually, yes, back in that day at that time, it was quite intense pain because I was really thinking of everything that I was going through, and it was pretty stressful. So, I was in a lot of pain back then—yes. It’s just that I got more nervous, sometimes when you need to go to court and those places make you—a lot of questions and it’s really stressful. It just made me feel more nervous and more anxious than normal and sometimes that affected my pain that I have. (Participant 22)

### Predisposing

#### Trust in Health Care Systems, Health Care Clinicians, and Trusted Environments

Many participants expressed trust in health care clinicians and in the associated environment as being central to their acceptance of nonopioid analgesics and trigger point injections. Participants noted that they found medications like nonopioid analgesics more acceptable when they were prescribed by a physician, reflecting their trust in health care clinicians. Participant 11 said, “Pills, if they are prescribed by a doctor then I take them.” Participant 22 said, “Whatever a doctor gives me, I will take it and if it’s not from my doctor, I will not do it.” Participant 7 said, “Yes, [the pills] do help me also. I did see a doctor. The doctor did prescribe me some pills that were helping me, but the doctor is no longer there.” Highlighting the necessity of professional guidance, participant 24 said, “It is necessary always following the doctor’s instructions and not self-prescribing yourself.”

A few participants iterated the importance of being in a safe and trusted environment. Participant 13 said, “I stopped going to the hospital (in home country) itself because of local discrimination stuff…. For me, over here now, to be completely honest. I think I can go somewhere, and I don’t have to worry.”

#### Concerns About Pain Treatment

Although participants generally desired pain relief, several expressed concerns about the effectiveness of treatments. The potential for addiction shaped their hesitations toward nonopioid analgesics and trigger-point injections.

##### Effectiveness of Pain Treatment

Participants described a range of experiences with pain treatments. Several noted that while nonopioid analgesics provided some relief, the effects were often short-lived. For example, participant 11 said, “Pain medication is just for calm the pain down for a while because after 6 or 8 hours after, the pain comes back.” Others described the delayed or partial relief and participant 15 explained, “They are good. But I would prefer injections…the pills don’t work instantly. They work in half an hour or a bit more.” Participant 22 acknowledged the variability in effectiveness and stated, “Some are useful, and some are not.” This sentiment extended beyond nonopioid analgesics: for example, participant 6, had previously tried physical therapy and stated, “I was going through the therapy…But then after when I go back to work and stand the pain comes back.”

##### Experience or Encounter With Addiction

Concerns about addiction to pharmacological treatment modalities (ie, nonopioid analgesics and trigger-point injections) were commonly cited. Participant 12 expressed fears that if she began taking injections, then she “would have to take injections every time [she has] the pain so it would go away.” A few participants attributed their fear of dependence to past experiences. Participant 20 said, “I do not like taking pills. Before, when I was detained with migration, I would take a lot, and I don’t want to become addicted to these pills.” Similarly, participant 2 observed that “in China, some pain relief medication may make the patient…get addicted.”

#### Language and Cultural Differences

Some participants expressed feeling more at ease when seeing a physician who shared their cultural background or spoke their language. Participant 10 stated that, by seeing someone who is part of the community, they would feel as if they could “speak freely.” Participant 16, noted that using an interpreter would be a barrier to effective communication and expressed frustration: “You can’t explain normally what’s the problem…how [to] explain that pain.”

#### Immigration Status

The majority of the participants had ongoing asylum cases. A few described how their asylum status impeded treatment access. Participant 3 shared that health care clinicians often requested missing documents, and said, “They are asking whenever I seek medical care…they ask me for a lot of documents which I don’t have.” While the participant did not specify which documents were requested, her broader narrative referenced expired insurance and unresolved legal work authorization, suggesting confusion about the documentation required for accessing health care.

### Enabling

#### Perceived Inadequate Insurance Coverage

Although most participants were insured, many reported uncertainties around insurance coverage that contributed to fears of high financial costs. Specifically, multiple participants were unsure how insurance policies provided for specialist care vs emergency treatment. Participant 18 was covered by New Jersey’s publicly funded insurance program and acknowledged that his insurance covered “primary care and emergencies” and did not think he could access specialized care. Participant 2 expressed being unsure about whether emergency services would be covered by his “white card.” Participant 8 expressed uncertainty about his insurance status and stated that “I work under this company’s payroll, but I don’t know if they give me some insurance because of that.” The gap in understanding insurance coverage could prevent participants from accessing certain pain treatments like trigger-point injections, which require specialist referral.

#### Financial and Work-Related Barriers

##### Need to Financially Support Dependents

Most participants had someone who was financially dependent on their current income. Participant 8, an individual with back pain who was instructed to rest, explained that “Right now my family depends on me. If I don’t work, then I’ll bring no food home.” Participant 14 stated that she had to “work through...and [she] can’t be without work” even when she felt sick and had to ask her daughter to come help her after school.

##### Unstable Work Conditions

Some participants described unpredictable hours and participant 14 noted that her employers only gave her 2 days off while she worked “20 months straight with them.” The limited time off prevented participants from seeking health care. In describing her dilemma around how to spend her 1 day off, participant 20 stated that “[she doesn’t] know which would be better if at home or at the doctor’s office.”

#### Logistical Challenges

Of the modalities investigated, physical therapy was viewed most positively, and the majority of participants indicated they would accept some form of physical therapy. However, attending 3 to 4 sessions weekly was burdensome for some. Participant 10 said that “2 days in a week is ok.” Travel further hindered uptake of physical therapy. As participant 1 explained that “Every time I come here, I need my wife to submit leave application and then take me here. So, it can’t be too frequent.” Some participants noted that balancing work and personal responsibilities with physical therapy can be challenging.

Participants who highlighted time and travel barriers expressed interest in home-based physical therapy as an alternative. Participant 18 expressed that “It’s much easier, because here traveling, moving going to cost you like, 2 hours, 3 hours sometimes half day. Like today I came here so I’m not going to work half a day.” Despite logistical concerns, physical therapy remained a favored option for most participants, with only a few expressing disinterest due to time or travel constraints.

### Need

#### Pain Severity

During the interview, many participants rated their pain level as at least 5 of 10, with a few reporting scores of 8 of 10 and higher. A few also stated that their pain caused interference in their daily lives. Participant 6 described pain so severe that “To get up in the morning, I can’t put my foot down.” Participant 8 noted that his pain “Started hurting more and more to the point that I’m not able to sit up or stand up easily.”

#### Strong Desire to Alleviate Pain

All participants expressed a willingness to engage with at least 1 type of intervention. These interventions for their chronic pain include physical therapy, nonopioid analgesics, and trigger-point injections.

##### Open to All Treatments

Driven by a strong desire to alleviate discomfort, most participants overwhelmingly expressed a willingness to try all 3 proposed treatment modalities. Participant 6 stated being willing to try “Anything that will help me,” and participant 14 emphasized being open to “Everything that might take my discomfort away.”

##### Conditional Acceptance Based on Pain Severity

Participants’ acceptance of pharmacological treatments (ie, nonopioid analgesics and trigger-point injections) was often conditional on pain severity. Participant 11 shared, “I’m kind of afraid of injection. But I mean if I have to, well I’ll have them.” Participant 10 said, “I try to avoid. I would like to avoid [nonopioid analgesics] but again, if there is a pain…then I can.”

#### Stress of Legal Status

Most participants expressed that their legal status caused them immense stress, affecting their pain symptoms and severity. Participant 21 reflected that “by being depressed, I realize, it triggers pain.” Participant 23 noted that his typical pain level was “usually 5 to 6” and surged to “8 or 9” during particularly stressful periods, while participant 16 described the onset of new pain, such as “neck pain and back pain,” that he associated with stress.

## Discussion

In this qualitative study of 25 refugee torture survivors with chronic somatic pain, we found that all participants expressed interest in at least 1 of the 3 standard treatment modalities for pain (ie, nonopioid analgesics, trigger-point injections, and physical therapy). This acceptability was shaped by several predisposing and need factors, including trust in the health care system and a strong desire to alleviate pain. Nevertheless, participants reported multiple perceived barriers that impeded uptake of acceptable therapies. These barriers include predisposing factors (eg, language barriers, cultural differences, and immigration status), enabling factors (eg, perceived inadequacies in insurance coverage, financial and work-related constraints, and logistical challenges), or need factors (eg, pain severity and the compounded stress of legal status). Although torture survivors with chronic pain are willing to engage in chronic pain treatment, our findings suggest that structural and systemic barriers limit their ability to do so.

Limited knowledge of the local health care system frequently hinders migrants from accessing health care.^20^ In the present study, uncertainties around insurance coverage constituted a major barrier to treatment accessibility. In accordance with similar trends among migrant workers and refugee populations in the US,^20,21^ we found that, although most participants had insurance, more than half were unsure about their coverage, which led to fears about high health care costs. Thus, a critical actionable item is the dissemination of knowledge about health care rights, which can empower refugees to navigate the system more effectively and to access necessary care. Additionally, greater social capital—including engagement with religious organizations or informal support networks—has been associated with improved insurance literacy and access to care.^21^ Our study did not explore social capital in this population, and future research should examine how community-based interventions and clinicians can enhance health care navigation among refugee populations.

Beyond structural barriers, legal stress emerged as both a barrier to treatment access and a contributor to pain. Chronic stress—legal stress in this population—exacerbates pain symptoms through various neurophysiological mechanisms.^22,23,24^ Participants frequently reported physiological and emotional responses to stress, including insomnia, muscle tension, and palpitations. Whereas associations between legal stress and mental health disorders in refugee populations have been established previously, the direct impact of legal stress on pain experience remains underexplored.^25,26,27,28^ A small qualitative study on chronic pain similarly described the impact of forced migration on pain experiences, although causality has yet to be established.^15^ The observed association between legal stress and pain is important and needs further attention.

In a cross-sectional study of Syrian refugees, the severity of chronic pain was significantly associated with the use of nonprescription painkillers.^29^ We similarly found that pain severity was a key factor for treatment acceptability. In our cohort of 25 torture survivors, many participants reported moderate pain levels that interfered with daily activities. Although most were open to treatment, concerns about pharmacological interventions, particularly regarding dependence, were common. Nevertheless, participants actively sought pain relief, primarily through over-the-counter, nonopioid analgesics. The preference for nonopioid analgesics, even among those hesitant about pharmacological treatments, suggested that accessibility strongly influences treatment choice.

Consistent with our findings, prior research in the general population has shown that higher pain severity is often associated with greater willingness to try nonpharmacological treatments.^30^ We observed a similar trend, with most participants expressing interest in physical therapy despite logistical constraints. To address these logistical barriers, home-based physical therapy programs may provide a feasible alternative for individuals facing unstable work conditions and competing priorities. Our findings highlight that limited pain treatment use stems from structural barriers that restrict access to effective care.

### Limitations

This study has several limitations. Although self-reported data can be subject to recall bias, our interview focused on recent, salient experiences, likely minimizing such bias. Our sample was predominantly male and primarily of Latin and Caribbean origin, which may limit the generalizability of our findings. Additionally, those who agreed to participate might differ systematically from those who did not, potentially influencing the range of experiences captured. Notwithstanding these limitations, our findings offer important exploratory insights into chronic somatic pain treatment in refugee torture survivors.

## Conclusions

In this qualitative study of 25 refugee torture survivors, we highlight the complex interplay between pain treatment acceptance and systemic barriers to care. Although use of over-the-counter remedies and willingness to engage in treatment was high, logistical, financial, and psychosocial obstacles hindered access to more effective, long-term therapies for chronic pain. Addressing these barriers through home-based treatment options and improved health care navigation resources is essential for facilitating equitable and sustained health care engagement in this population.

## Notes

Supplement.
Data Sharing Statement
