# Supplementary material for: Acceptability and Barriers to Chronic Pain Treatment in Refugee Torture Survivors
Source: JAMA Netw Open. 2025 Aug 28;8(8):e2529775. doi: 10.1001/jamanetworkopen.2025.29775 (PMC12395311; doi:10.1001/jamanetworkopen.2025.29775)
Supplement: Supplement. — Data Sharing Statement [file jamanetwopen-e2529775-s001.pdf]

Data Sharing Statement

Virk. Acceptability and Barriers to Chronic Pain Treatment in Refugee Torture Survivors. *JAMA Netw Open*. Published August 28, 2025. doi:10.1001/jamanetworkopen.2025.29775

Data
Data available: No
